# The Pharmaceutical Era for May

**Published:** 1894-07

**Authors:** 


THE PHARMACEUTICAL ERA FOR MAY.

The May 1st issue of the *Pharmaceutical Era* is called its "MOVING DAY NUMBER," and commemorates the removal of that paper from Detroit to New York. We understand that the copies of this issue are the most elaborate ever sent out by any drug publication in this country, and the edition is said to be the largest ever issued by that class of papers.

Over the regular cover of the journal has been placed a lithographed cover showing a moving scene on the front page, and a conspicuous New York view on the back cover. The reading pages of the issue have been materially increased, and are embellished throughout by attractive illustrations. Among the special articles is a sketch of the history and mechanical production of the *Era*, also an article on "A Druggist's Visit to New York." The advertising pages are very numerous, and the publication, as a whole, a credit to its publishers.

